# Supplementary material for: Unraveling Genetic Variation and Inheritance Patterns in Newly Developed Maize Hybrids for Improving Late Wilt Disease Resistance and Agronomic Performance Under Artificial Inoculation Conditions
Source: Life (Basel). 2024 Dec 5;14(12):1609. doi: 10.3390/life14121609 (PMC11677832; doi:10.3390/life14121609)
Supplement: Supplementary file 1 [file life-14-01609-s001.zip › life-3350986-supplementary.pdf]

Table S1. Description of the lines and testers used in this study.

Code	Name	Description	Source
IL-301	Inb.180	Line	Egypt
IL-302	Inb.174	Line	Egypt
IL-303	Inb.202	Line	Egypt
IL-304	Inb.205	Line	Egypt
IL-305	CML217	Line	Mexico
IL-306	CML224	Line	Mexico
IL-307	CML225	Line	Mexico
IL-308	CML226	Line	Mexico
IL-309	CML228	Line	Mexico
T1	SC-167	Tester	Egypt
T2	Pioneer-3062	Tester	Egypt
T3	TWC-360	Tester	Egypt
